# Tiptoe Walking in the Presence of Superimposed Aeromonas With Dengue Infection

**DOI:** 10.7759/cureus.46136

**Published:** 2023-09-28

**Authors:** Sri Arun Sellvam, Shashank Raghu, Juzaily F Leong, Firdaus Hafni, Mohd Yazid Bajuri

**Affiliations:** 1 Orthopedics and Traumatology, Universiti Kebangsaan Malaysia Medical Center, Kuala Lumpur, MYS; 2 Orthopedics and Traumatology, Hospital Pulau Pinang, George Town, MYS

**Keywords:** tip-toe, limb salvage surgery, septicaemia, dengue, : aeromonas hydrophilia

## Abstract

The rise in limb salvage surgeries has led to an increase in post-traumatic equinus contractures, resulting in secondary causes of tiptoe walking. There is notable evidence linking superimposed bacteremia to heightened risks of morbidity and mortality in dengue patients, particularly when the immune system is compromised, leading to documented cases of *Aeromonas*-induced necrotizing fasciitis with myonecrosis. A case report from August 2019 involves a five-year-old boy admitted for dengue fever, which progressed to severe bilateral leg necrotizing fasciitis caused by concurrent *Aeromonas* infection. The child further underwent urgent surgical debridement and post-operatively was diagnosed with necrotizing fascio-myositis of the bilateral leg. Despite urgent surgical intervention, the patient developed long-term complications of persistent equinus contractures in both legs, necessitating subsequent surgical Achilles tendon lengthening. This case underscores the potential fatality of superimposed *Aeromonas septicaemia* in dengue patients and emphasizes the need for vigilant monitoring and intervention in serious soft tissue infections in growing children and adolescents.

## Introduction

The accomplishment of motor milestones like standing and walking is looked forward to and celebrated by parents. Medical consultation and anxiety are frequently triggered by deviation from the perceived norm. Despite the fact that the majority of these variances are within the normal physiological range, it is important to identify pathological processes and abnormal development to help with the management [1]. Accordingly, tiptoe walking, referred to as toe walking, is a walking pattern in which the heel does not contact the ground. Tiptoe walking is primarily divided into two categories: early onset and late onset [2,3]. Achilles contractures are another significant cause of tiptoe walking; hence, it is necessary to assess the severity of static equinus contractures further down [2,3].

Significant evidence links superimposed bacteremia to increased risk of morbidity and mortality in dengue patients [4]. *Aedes aegypti* is one of the reasons causing *Aeromonas* bacteremia since the *Aeromonas* species is a part of the midgut microbiota of the dengue vector. When the host's defense is weak, necrotizing fasciitis with myonecrosis caused by *Aeromonas* manifestation has been documented [5].

We encountered a case of necrotizing fasciitis with myonecrosis on both legs caused by a dengue infection with concurrent bacteremia of *Aeronomas* leading to long-term complications of Achilles tendon contraction, causing the child to walk on tiptoes due to significant static equinus contracture.

## Case presentation

In August 2019, a five-year-old boy arrived at the emergency department with vomiting, epigastric pain for three days, and a persistently high temperature. The boy was admitted to the hospital for dengue fever with warning signs after a positive NS1 test result revealed the presence of dengue. Repetitive temperature spikes despite regular antipyretic led to further suspicions as the onset of disorientation and shortness of breath set in. The child's condition deteriorated to dengue fever with compensatory shock.

On day 12 after admission, the patient developed pleural effusion and severe swelling in both calves despite stable vital signs. Blood culture and sensitivity test findings showed the presence of *Aeromonas hydrophilia*. The child started on a course of antibiotics with cefepime and ciprofloxacin according to sensitivity from culture results. The condition of the bilateral leg, which was initially diagnosed as calf swelling from intramuscular edema, got worse with ecchymosis and increasing swelling.

Considering anticipated compartment syndrome, the case was further referred to the pediatric orthopedic unit. When evaluated, the neurovascular condition of the bilateral lower limbs was intact, although there were skin bullae and crepitus across the calf on both sides. A further diagnosis of necrotizing fasciitis in both legs was suggested. The child underwent an urgent surgical treatment of incision and drainage on the bilateral leg.

**Figure 1:**
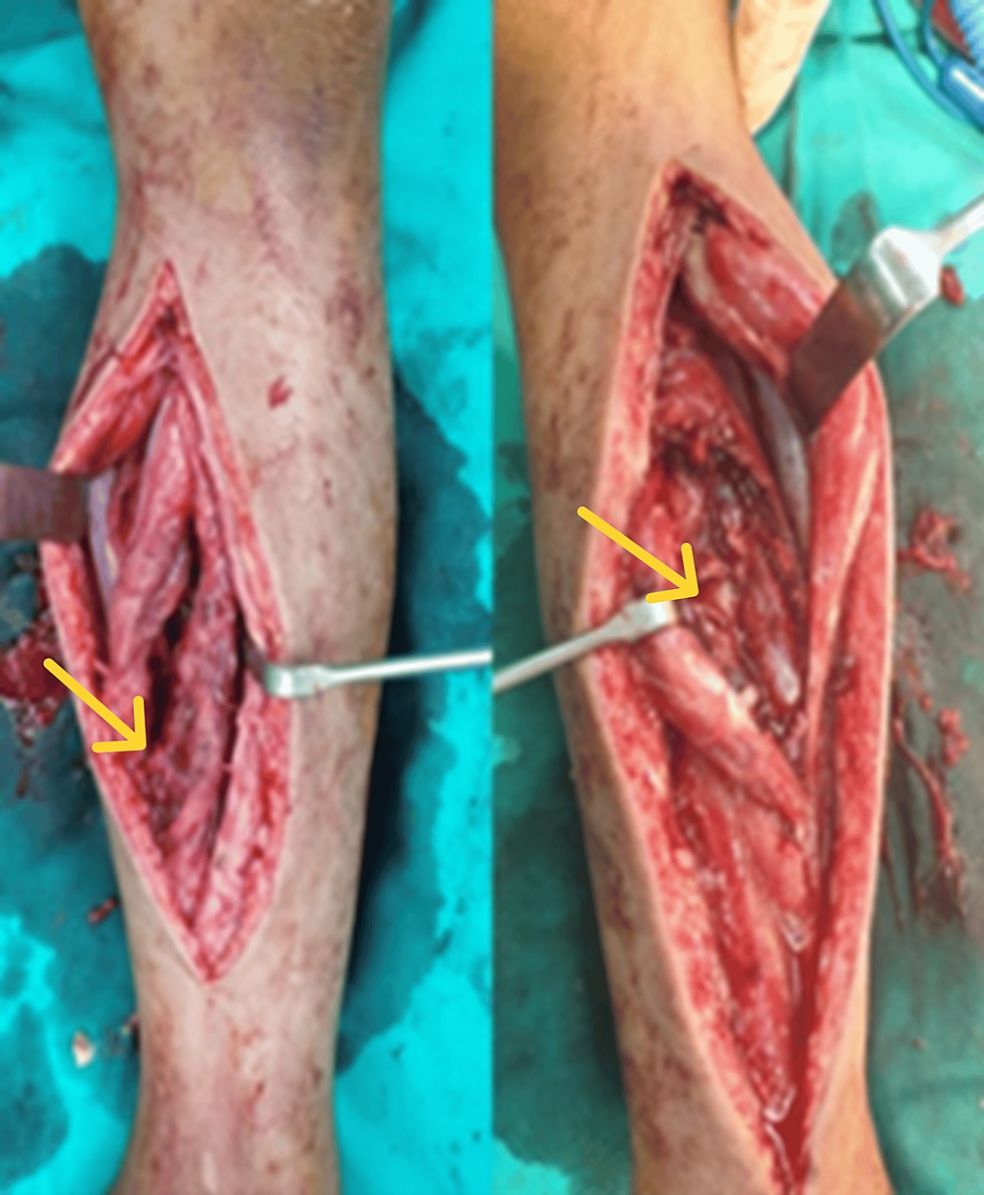
Intraoperative findings showing myonecrosis of the bilateral leg with necrotizing fasciitis

The results of the surgery revealed a dishwater-like fluid accumulation in each leg and the destruction of the fascia and muscles over the lateral and posterior compartments, supporting the diagnosis of necrotizing fascio-myositis (Figure 1). Tissue and fluid sensitivity tests performed intraoperatively revealed the presence of *Aeromonas hydrophilia*. Till the state of the wound improved, several debridement and washouts were performed. A moist dressing was applied to the wounds on both legs in addition to continuing the antibiotics.

**Figure 2:**
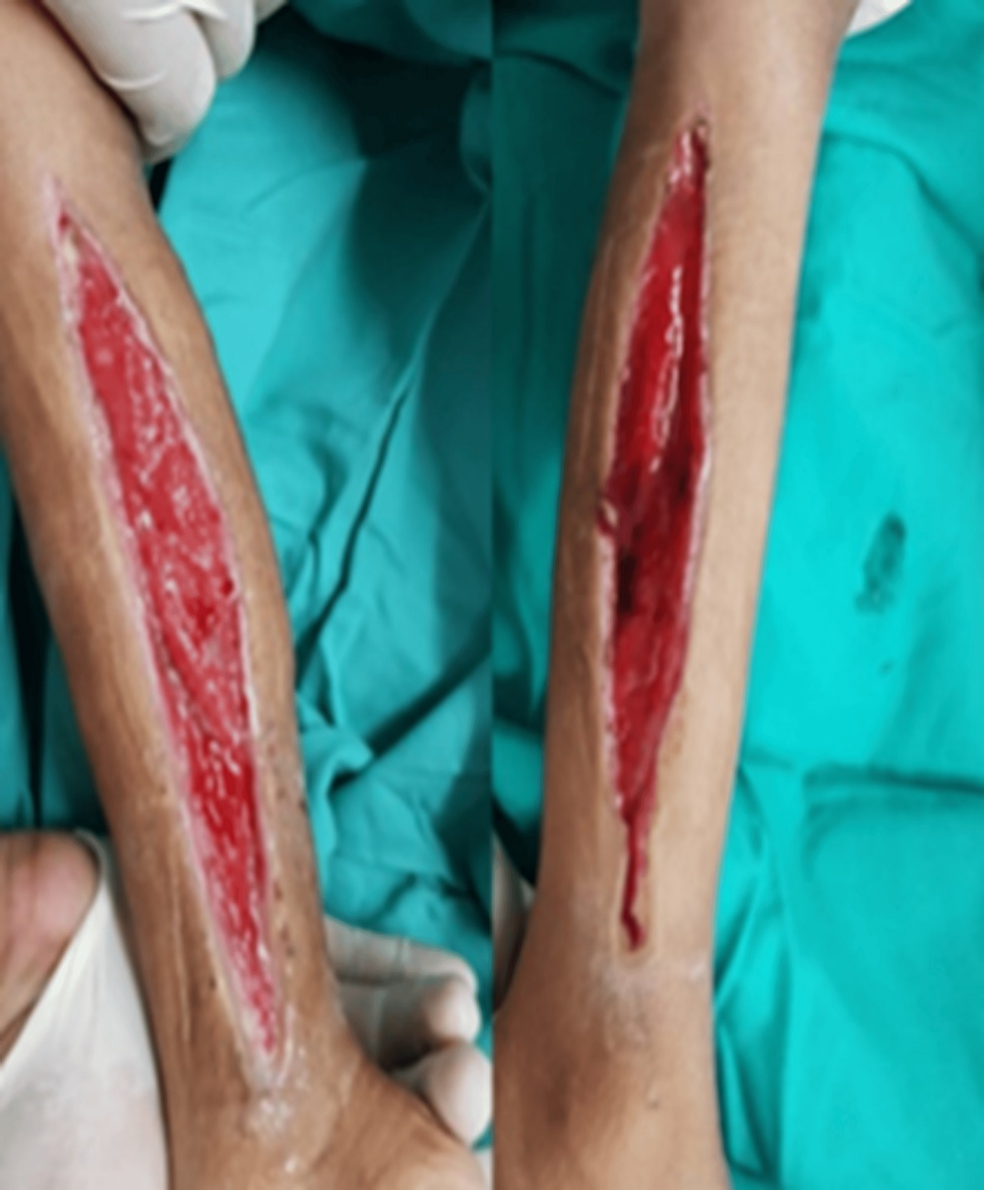
Post-operative wound condition in the second week prior to wound closure

The child was well, and the wound inspection was cleaned at the clinic two weeks after discharge (Figure 2). Later, the secondary suturing of the bilateral leg incisions was performed. When examined six weeks after surgery, the youngster was able to walk effectively. Early physiotherapy started and continued for the next three months. No subsequent follow-up was given after that as the child was well and active.

**Figure 3:**
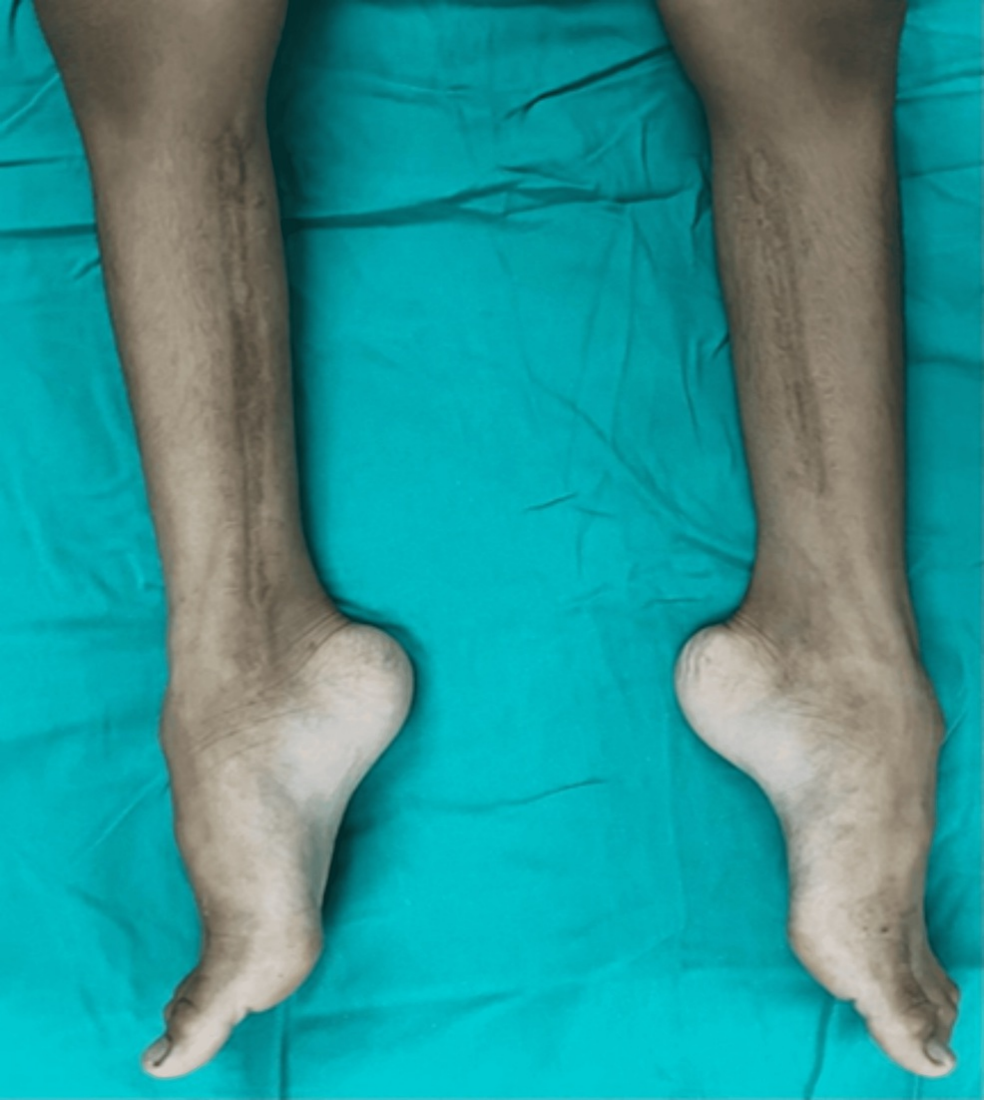
Post-operative three years showing static equinus contracture of the bilateral leg with tight Achilles tendon

**Figure 4:**
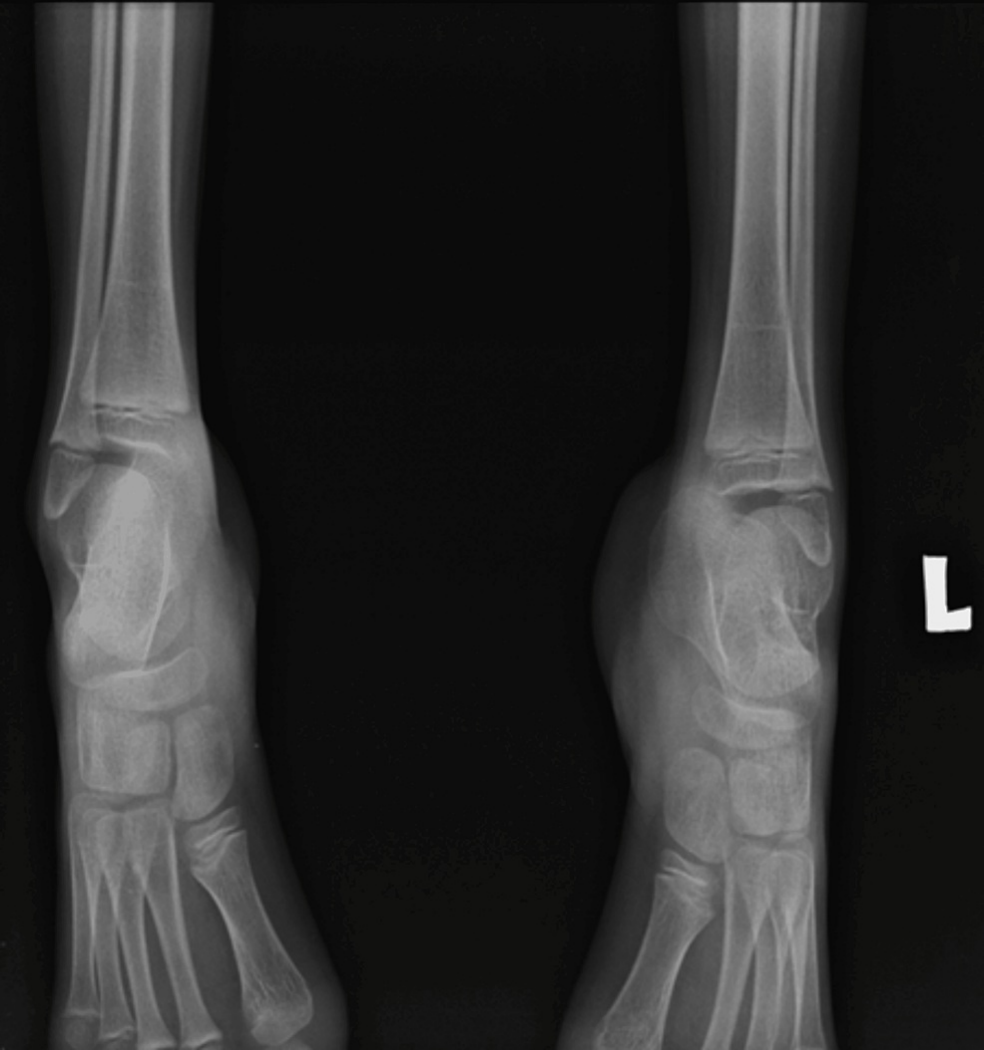
Radiographical images of the bilateral foot showing no bony deformity of the bilateral foot

The child resurfaced three years later, in February 2022, walking on tiptoes and displaying static equinus contracture in both legs. Upon inspection, it was discovered that both legs had muscle atrophy and stiff Achilles tendons (Figure 3). Radiographical images of the bilateral foot showed no bony deformity of the bilateral foot, indicating the soft tissue contracture severity (Figure 4).

The patient underwent Z-plasty and adhesiolysis surgery for bilateral Achilles tendon lengthening. After surgery, plantigrade at 0 degrees for the right ankle and dorsiflexion at 10 degrees for the left ankle was achieved. Post correction, both ankles fell within the normal range for ankle joint dorsiflexion at non-weight bearing. Additionally, a bilateral above-knee fiberglass cast was applied to the child for six weeks. Upon further follow-up at the clinic after six weeks, neurovascular status was intact, and the wound was well healed. Measurement of both ankle dorsiflexion at three months showed improvement of right ankle dorsiflexion at 5 degrees and left ankle dorsiflexion at 10 degrees similar to post-operative correction. The child was able to plant his foot well on the ground and able to ambulate without pain on further follow-up at six months.

## Discussion

The first stage in assessment is to decide if tiptoe walking is an early-onset or late-onset disorder. The second part of the initial assessment involves gathering comprehensive family history information and identifying the underlying neuromuscular issues [3]. Early-onset tiptoe walking is when a child first starts walking on his or her toes a few months after becoming independent. The two differential diagnoses for early-onset tiptoe walking that are most frequently used, with a few rare exceptions, are mild spastic diplegic cerebral palsy and idiopathic tiptoe walking [6].

The phrase "late-onset tiptoe walking" describes the transition from the established regular heel-toe stride to tiptoe walking [7]. It is recommended to seek prompt evaluation by a neurologist as this is most often caused by a neuromuscular deficiency in order to rule out spastic diseases, anomalies of the spinal cord, peripheral neuropathies, or muscular dystrophies [1].

Eventually, both early-onset and late-onset tiptoe walkers could develop gastrocsoleus contractures that are static or dynamic. The success of limb salvage surgery has led to an increase in the prevalence of post-traumatic equinus contractures leading to other secondary causes of tiptoe walking. Soft tissue defects and post-traumatic contractures of the foot and ankle present challenging cases for orthopedic and plastic surgeons [8].

Assessment of Achilles contracture is primarily done for therapeutic purposes following diagnosis [1]. Therefore, to accurately measure a contracture, one should measure the ankle dorsiflexion while the knee is fully extended and the talonavicular joint (midfoot) is stable in its anatomic position. This is due to the gastrocnemius muscle's posterior knee joint crossing. If the midfoot is allowed to pronate due to the forefoot's relative pronation to the hindfoot, the measurement of ankle dorsiflexion will be elevated incorrectly by 5 to 10 degrees [2,3].

Regardless of their origin, permanent equinus contractures typically require medical attention and should be sent to an orthopedic specialist. The aim of surgical treatment for gastrocsoleus contracture is usually dorsiflexion restoration to 5 degrees [1,2]. Achilles tendon lengthening is primarily required for equinus contractures. The reversed sural fasciocutaneous island flap has gained popularity as a good substitute for free tissue transfer for the soft tissue coverage of the distal third of the leg, ankle, and foot in severe cases with soft tissue defects. Shu et al. reported a progressive correction technique using the hinged Ilizarov in conjunction with a reversed sural fasciocutaneous island flap transfer for the soft tissue coverage of the heel for equinus foot deformity, which produced positive outcomes [8].

In our situation, the child was treated successfully from the initial admission and was discharged from our care early. Considering the extent of the soft tissue damage to the lateral and posterior compartments of both legs, the growing child should have been carefully monitored and provided with the required ankle-foot orthosis to prevent or lessen the expected equinus contracture.

Superimposed bacteremia has increased the risk of morbidity and mortality in dengue patients, although it is underreported. *Aeromonas* species is one of the midgut microbiota of the dengue vector *Aedes aegypti*, making it one of the sources of *Aeromonas* bacteria [5]. Aeromonas is an anaerobic gram-negative bacillus found naturally in aquatic environments [6]. There is a risk of *Aeromonas* bacteremia from contaminated tap water or undercooked fish soup.

Generally, cellulitis, myonecrosis with necrotizing fasciitis, and ecthyma gangrenosum are common skin and soft tissue infections in humans that can be caused by *Aeromonas hydrophila* [6]. Endotoxins from *Aeromonas*, however, have the potential to produce myonecrosis with necrotizing fasciitis and ecthyma gangrenosum in immunocompromised patients. These conditions can be deadly or extremely disabled and even result in amputation [9].

One class of antibiotics that consistently combat *Aeromonas* species is third-generation cephalosporins. Recent research suggests that ciprofloxacin or azithromycin should be used as the first-line medications for people who have *Aeromonas* infections [9].

## Conclusions

The pathogenicity of *Aeromonas* emphasizes the severity and rapid spread of infections caused by it. Involvement of muscles, indicating advanced necrotizing fasciitis, is linked to poor prognosis and survival rates. In dengue patients, concurrent bacteremia such as *Aeromonas septicaemia* carries a potential for fatality. It underscores the importance of consistent monitoring for young individuals with serious soft tissue infections that require surgical action. Early surgical intervention is necessary to prevent deformities, and the importance of long-term follow-up in a growing age group cannot be overstated. The case discussed highlights the necessity of ongoing physical therapy for rehabilitation in growing individuals as well as parental counseling to detect any early deformity presentation.
